# Medicolegal aspects of digestive endoscopy: Results of a Chilean national survey

**DOI:** 10.1055/a-2570-6490

**Published:** 2025-05-12

**Authors:** Oscar Corsi, María Jesús Fuenzalida, José Ignacio Vargas, Verónica Silva, Maximiliano Figueroa, Juan Andrés Prato, Andrea Künsemüller, Alberto Espino

**Affiliations:** 1 28033 Department of Gastroenterology, School of Medicine, Pontificia Universidad Catolica de Chile, Santiago, Chile; 2 Endoscopy Unit, Hospital Clínico Red UC-CHRISTUS, Santiago, Chile; 3 37619 Department of Gastroenterology, Universidad de Chile Facultad de Medicina, Santiago, Chile; 4 60765 Instituto Chileno-Japonés de Enfermedades Digestivas, Hospital Clínico San Borja Arriarán, Santiago, Chile; 5 Endoscopic Surgery Unit, Hospital Regional Libertador Bernardo O’Higgins, Rancagua, Chile; 6 28033 Department of Psyquiatry, School of Medicine, Pontificia Universidad Catolica de Chile, Santiago, Chile; 7 Fundación de Asistencia Legal del Colegio Médico de Chile A.G. (FALMED), Santiago, Chile

**Keywords:** Quality and logistical aspects, Quality management, Performance and complications, Training

## Abstract

**Background and study aims:**

Medical professional liability (MPL) is a significant concern for gastrointestinal physicians, yet there are limited data available from Latin America. We aimed to assess frequency of complaints and lawsuits related to digestive endoscopy among gastrointestinal endoscopists in Chile and to identify associated factors.

**Methods:**

An online survey collected sociodemographic data, information about endoscopy unit characteristics, and MPL-related experiences. Invitations were sent to 525 gastrointestinal endoscopists in Chile between August and September 2022. Associations between categorical variables were analyzed using the Chi-square test.

**Results:**

In total, 140 endoscopists participated (response rate: 26.7%). Mean age was 48.8 years; 68.6% were gastroenterologists, 70.7% were male, and 95% had MPL insurance. Written complaints were reported by 55% of participants, with an average of 1.5 complaints per year. The most common causes were procedure costs, adverse events (AEs), and sedation issues. Colonoscopy was the procedure most frequently associated with complaints (63.2%). Complaints related to AEs included perforation (48.7%), hemorrhage (23.7%), pancreatitis (21.1%), and death (13.2%). Factors associated with complaints included years of endoscopic practice ( *P* = 0.047), therapeutic procedures ( *P* < 0.001), and patient satisfaction assessments ( *P* = 0.048). Of respondents, 14.5% reported at least one lawsuit. Factors associated with lawsuits included age ( *P* = 0.0047), male gender ( *P* = 0.0033), Chilean nationality ( *P* = 0.0257), therapeutic procedures ( *P* = 0.004), and patient satisfaction assessments ( *P* = 0.002).

**Conclusions:**

Gastrointestinal endoscopists are frequently exposed to complaints and lawsuits. Key factors include procedure costs, AEs, sedation practices, years of experience, type of endoscopic procedure, and communication. Proactive strategies to address these factors could mitigate medico-legal risks and improve patient outcomes.

## Graphical Abstract

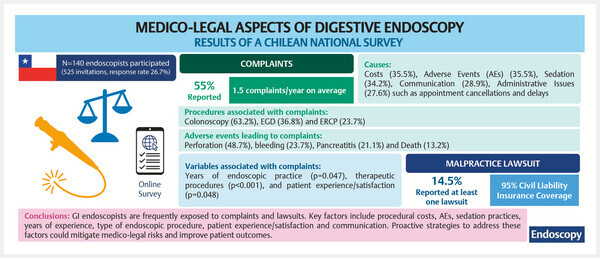

## Introduction

Medical professional liability (MPL) refers to the legal obligation of healthcare professionals to adhere to accepted standards of care and skill within their specialty. For many physicians, MPL is an inevitable aspect of their clinical careers, with gastroenterologists among the medical specialties most frequently involved in litigation in the United States 1 2 3 . Procedure-related MPL complaints represent approximately 25% of all gastrointestinal-related complaints. Among these, 52% involve colonoscopy, 16% endoscopic retrograde cholangiopancreatography (ERCP), and 11% esophagogastroduodenoscopy (EGD). Although generally safe, EGD and colonoscopy involve a risk of rare but potentially serious adverse events (AEs) 4 .

In the United Kingdom, although most endoscopy-related complaints are resolved locally by National Health Service Trusts, approximately 4% of cases referred to the NHS Litigation Authority (NHSLA) progress to court. Between 1995 and 2008, the NHSLA handled 418 endoscopy-related negligence claims, representing 17% of all NHS claims requiring court settlement. EGD and colonoscopy were associated with 65% of settled claims, but ERCP was the procedure most frequently linked to claims related to a fatal outcome. Factors underlying claims following fatal complications included: inappropriate endoscopic treatment/direct complication; delay in recognizing complication and its management; consent issues; and medication issues, including incorrect management of anticoagulation 5 .

In the United States, litigation patterns related to colon cancer screening and colonoscopy emphasize delays in diagnosis and treatment as significant grounds for complaints and lawsuits 6 7 . Such litigation influences clinical decisions, because physicians with a history of legal cases may adopt more aggressive follow-up strategies in patients with Barrett’s esophagus 8 .

An analysis of 18 malpractice cases in Japan found that 44% were related to EGD, 22% to colonoscopy, 22% to endoscopic sphincterotomy, and 11% to ERCP. Of these cases, 94% were due to complications, whereas 6% involved misdiagnosis. In 10 cases, complications resulted in patient death 9 .

The European Society of Gastrointestinal Endoscopy (ESGE) has responded to these trends with updated guidelines emphasizing the importance of informed consent, patient-centered communication, and comprehensive documentation to enhance patient safety and reduce legal risks. These guidelines advocate for shared decision-making tailored to individual patient needs and preferences, thereby supporting a high standard of patient-focused care 10 .

Despite growing data from various regions, available information on MPL related to gastrointestinal endoscopy from Latin America is scarce.

This study aimed to evaluate the frequency of complaints and lawsuits related to digestive endoscopy among gastrointestinal endoscopists in Chile and identify factors contributing to these medicolegal issues, based on data collected from a national survey of endoscopists.

## Methods

An observational study was conducted using an online survey administered to endoscopists practicing in Chile between August and December 2022. The survey was developed by the research team (OC, MF, AE) based on international recommendations 11 12 13 14 15 16 17 , and it covered demographic information, professional training, management of endoscopy units, and medicolegal situations. The survey also inquired about causes of complaints and lawsuits. The questionnaire was initially reviewed by the Board of the Chilean Association of Endoscopy (ACHED) of the Chilean Society of Gastroenterology (SCHGE), the Department of Endoscopic Surgery of the Chilean Society of Surgeons (SOCICH), and a representative from the Legal Department of the Medical Association of Chile A.G. (FALMED). The survey is available as Supplementary Material. The survey was tested as a pilot study in 10 endoscopists to review the format, clarity, applicability of the questions, and platform functionality.

An invitation to participate in the survey was sent to 525 endoscopists registered in the SCHGE and the Department of Endoscopic Surgery of the SOCICH by email and social media. The criterion for receiving an invitation was to be a member of one of these organizations. The sole inclusion criterion in the study was agreement to the informed consent form. Ethics approval was obtained from the Ethics Board of the Pontificia Universidad Católica de Chile (ID 220907002). All participants provided informed consent before participating in the study. All responses were anonymous.

### Definitions

Basic therapeutic endoscopy practice was defined as performing polypectomy, hemostasis, foreign body extraction, and percutaneous endoscopic gastrostomy placement. Advanced therapeutic endoscopy included ERCP, endosonography, stent placement in any segment, deep enteroscopy, endoscopic mucosal resection, endoscopic submucosal dissection, and per-oral endoscopic myotomy (POEM).

Chilean legislation establishes various rights and duties for patients 18 . According to Chile’s Patients’ Rights and Duties Law, if a patient believes that a healthcare provider has violated their rights, they may file a direct written complaint with the provider, seeking either compensation or corrective action. If the patient’s request is not satisfactorily addressed, they may escalate the issue to health authorities, such as the Superintendency of Health or the Regional Health Authorities, which have the power to investigate and impose administrative sanctions, as well as invite the parties to mediation; however, they do not have the authority to order financial compensation.

In contrast, a medical malpractice lawsuit is a civil case filed in court to seek compensation for harm caused by negligence. In Chile, before filing a medical malpractice lawsuit, the law mandates mediation. Mediation is intended to foster a mutually acceptable resolution without resorting directly to court proceedings. If mediation fails, the patient may proceed to file a lawsuit in a civil court.

Finally, in Chile, MPL insurance for medical practitioners is a type of insurance designed to cover civil liability of healthcare professionals for damages caused to third parties in the course of their professional duties. This insurance protects the medical professional, as well as other healthcare workers, against compensation arising from acts or omissions that have caused harm to patients, whether due to negligence, errors, or failures in medical care.

### Statistical analysis

The following independent variables were proposed as factors related to self-reported complaints and self-reported malpractice lawsuits: age, medical specialty, years of endoscopic practice, advanced endoscopic practice, hospital or outpatient unit, university clinical training field, unit funding, existence of a recording system for an AEs, follow-up after polypectomy, follow-up after occurrence of an AE, personnel responsible for the informed consent process, and type of informed consent.

Categorical variables were expressed as relative frequencies and percentages, and continuous variables as means, standard deviations (SD), medians, and ranges as specified in the text. The Chi-square test or Fisher's exact test was used to assess the association between categorical variables, depending on the number of observations. Two-sample mean difference test was used to compare mean age according to self-report of complaints or malpractice lawsuits. Statistical analyses were conducted using the R statistical software (version 4.3.1, R Foundation for Statistical Computing, Vienna, Austria). Statistical significance was set at *P* < 0.05.

## Results

### Survey participation and demographics

One hundred and forty responses were obtained with a participation percentage of 26.7%. Mean age of respondent was 48.8 years old and 70.7% of participants were male. Most respondents reported gastroenterology as their specialty (68.6%), had completed a formal training program (94.3%), and had more than 10 years of practice (57.9%). In addition, 31.2% held a leadership role in their endoscopy unit and 31.4% performed advanced endoscopic therapy.

### Self-reporting of complaints

**Fig. 1:**
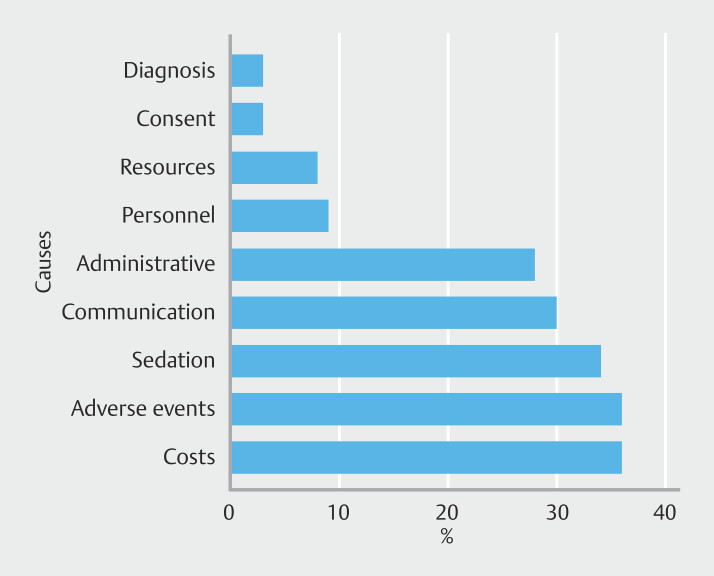
Most frequent causes of complaints identified by participants in the study.

**Fig. 2:**
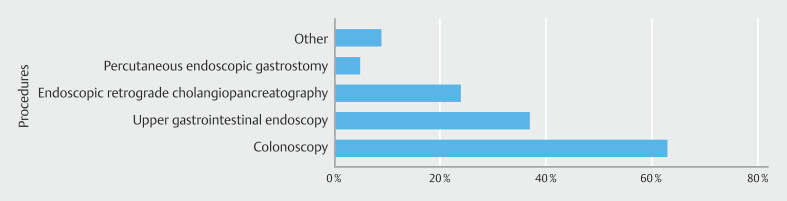
Most frequent procedures associated with complaints identified by participants in the study.

Table 1 shows the association between general characteristics of participants and self-reports of complaints. Of the participants, 55.0% have faced a complaint associated with endoscopic practice. Among those who have faced complaints (n = 77), the mean number per year was 1.51 (1.21) (range 1–8 complaints/year). The most frequent causes were procedure costs (35.5%), occurrence of an AE (35.5%), sedation issues (34.2%), communication problems (28.9%), and administrative issues (27.6%) such as appointment cancellations or delays ( Fig. 1 ). Procedures associated with complaints were colonoscopy (63.2%), EGD (36.8%), and ERCP (23.7%) ( Fig. 2 ). The most reported AEs leading to complaints were perforation (48.7%), bleeding (23.7%), pancreatitis (21.1%), and death (13.2%). Years of endoscopic practice ( *P* = 0.047), type of endoscopic procedures performed ( *P* < 0.001), and measurement of patient experience and/or satisfaction at the endoscopy center ( *P* = 0.048) were the only work unit variables associated with self-report of malpractice complaints ( Table 1 ).

**Table:** **Table 1** Association between general characteristics of participants and self-reported complaints.

	Complaints		
	No (n = 63)	Yes (n = 77)	*P* value
Age			
Mean (SD)	47.8 (11.8)	49.7 (10.7)	0.321
Median [Min, Max]	45.0 [31.0, 75.0]	47.0 [34.0, 76.0]	
Missing	0 (0%)	4 (5.2%)	
Gender			
Female	24 (38.1%)	17 (22.1%)	0.0594
Male	39 (61.9%)	60 (77.9%)	
Medical specialty			
Surgery	15 (23.8%)	18 (23.4%)	0.938
Gastroenterology	44 (69.8%)	52 (67.5%)	
Other	2 (3.2%)	4 (5.2%)	
Pediatrics	2 (3.2%)	3 (3.9%)	
Nationality			
Chilean	60 (95.2%)	70 (90.9%)	0.538
Ecuadorian	1 (1.6%)	0 (0%)	
Venezuelan	2 (3.2%)	4 (5.2%)	
Bolivian	0 (0%)	1 (1.3%)	
Colombian	0 (0%)	1 (1.3%)	
Hungarian	0 (0%)	1 (1.3%)	
Years of practice			
Between 5 to 10 years	21 (33.3%)	20 (26.0%)	0.0467
More than 10 years	30 (47.6%)	51 (66.2%)	
Less than 5 years	12 (19.0%)	6 (7.8%)	
Have you completed a formal training program in digestive endoscopy?			
No	3 (4.8%)	5 (6.5%)	0.942
Yes	60 (95.2%)	72 (93.5%)	
Your role in the endoscopy unit: Select all the alternatives that correspond to your duties in the endoscopy unit			
Diagnostic	14 (22.2%)	4 (5.2%)	< 0.001
Advanced therapeutics	11 (17.5%)	33 (42.9%)	
Basic therapeutics	38 (60.3%)	40 (51.9%)	
Endoscopy unit			
Outpatient	7 (11.1%)	8 (10.4%)	0.942
Hospital	4 (6.3%)	6 (7.8%)	
Mixed	52 (82.5%)	63 (81.8%)	
Is your unit a university unit and/or is it an endoscopy training center?			
No	39 (61.9%)	47 (61.0%)	1.000
Yes	24 (38.1%)	30 (39.0%)	
Type of funding of your endoscopy unit			
Mostly private	30 (47.6%)	50 (64.9%)	0.059
Mostly public or governmental	33 (52.4%)	27 (35.1%)	
Do you apply surveys or other instruments to measure user experience and/or satisfaction in your endoscopy unit?			
No	37 (58.7%)	40 (51.9%)	0.0482
I do not know	13 (20.6%)	8 (10.4%)	
Yes	13 (20.6%)	29 (37.7%)	
Do you have a support team or professional responsible for the continuous improvement in your endoscopy unit or institution?			
No	30 (47.6%)	34 (44.2%)	0.28
I do not know	14 (22.2%)	11 (14.3%)	
Yes	19 (30.2%)	32 (41.6%)	
Are patients contacted before procedures to solve questions? (e.g. by phone calls, mobile applications, email or other)			
No	20 (31.7%)	20 (26.0%)	0.366
I do not know	4 (6.3%)	2 (2.6%)	
Yes	39 (61.9%)	55 (71.4%)	
Do you have local or standardized protocols for sedation and/or analgesia for endoscopic procedures?			
No	27 (42.9%)	32 (41.6%)	0.77
I do not know	4 (6.3%)	3 (3.9%)	
Yes	32 (50.8%)	42 (54.5%)	
Regarding anesthesia assistance during endoscopic procedures			
We have medical anesthesia equipment	46 (73.0%)	64 (83.1%)	0.214
We do not have medical anesthesia equipment	17 (27.0%)	13 (16.9%)	
Does your endoscopy unit get in contact with all patients who underwent polypectomy within 1 week after the endoscopic procedure to resolve questions and/or to detect complications?			
No	39 (61.9%)	62 (80.5%)	0.0052
I do not know	15 (23.8%)	4 (5.2%)	
Yes	9 (14.3%)	11 (14.3%)	
Does your endoscopy unit get in contact or follow up with patients who have an adverse event during an endoscopic procedure?			
No	17 (27.0%)	21 (27.3%)	0.444
I do not know	15 (23.8%)	12 (15.6%)	
Yes	31 (49.2%)	44 (57.1%)	
Who performs the informed consent procedure before an endoscopic procedure?			
Medical doctor	44 (69.8%)	57 (74.0%)	0.719
Other health professional	19 (30.2%)	20 (26.0%)	
Does your endoscopic unit report most adverse events by a recording system?			
No	5 (7.9%)	11 (14.3%)	0.431
I do not know	9 (14.3%)	8 (10.4%)	
Yes	49 (77.8%)	58 (75.3%)	
Regarding existing medical liability insurance and/or legal advice, which one do you have?			
None	3 (4.8%)	4 (5.2%)	1.00
FALMED insurance or other	60 (95.2%)	73 (94.8%)	

### Medical malpractice lawsuits

Table 2 shows the association between general characteristics of participants and self-report medical malpractice lawsuits. Of the participants, 14.5% reported at least one lawsuit related to an endoscopic procedure. Age ( *P* = 0.005), gender ( *P* = 0.003), Chilean nationality ( *P* = 0.026), and type of endoscopic procedure performed ( *P* = 0.004) were associated with the report of medical malpractice lawsuit.

In addition, patient experience and/or satisfaction at the endoscopy center ( *P* = 0.002) was the only work unit variable associated with this outcome ( Table 2 ).

**Table:** **Table 2** Association between general characteristics of participants and self-reported malpractice lawsuit.

	Malpractice lawsuit		
	No (n = 119)	Yes (n = 21)	*P* value
Age			
Mean (SD)	47.6 (10.9)	55.5 (10.9)	0.0047
Median [min, max]	44.0 [31.0, 76.0]	55.0 [40.0, 75.0]	
Missing	4 (3.4%)	0 (0%)	
Gender			
Female	41 (34.5%)	0 (0%)	0.0033
Male	78 (65.5%)	21 (100%)	
Medical specialty			
Surgery	26 (21.8%)	7 (33.3%)	0.269
Gastroenterology	84 (70.6%)	12 (57.1%)	
Other	4 (3.4%)	2 (9.5%)	
Pediatrics	5 (4.2%)	0 (0%)	
Nationality			
Chilean	111 (93.3%)	19 (90.5%)	0.0257
Ecuadorian	1 (0.8%)	0 (0%)	
Venezuelan	1 (0.8%)	0 (0%)	
Bolivian	6 (5.0%)	0 (0%)	
Colombian	0 (0%)	1 (4.8%)	
Hungarian	0 (0%)	1 (4.8%)	
Years of practice			
Between 5 to 10 years	38 (31.9%)	3 (14.3%)	0.066
More than 10 years	64 (53.8%)	17 (81.0%)	
Less than 5 years	17 (14.3%)	1 (4.8%)	
Have you completed a formal training program in digestive endoscopy?			
No	7 (5.9%)	1 (4.8%)	1.00
Yes	112 (94.1%)	20 (95.2%)	
Your role in the endoscopy unit: Select all the alternatives that correspond to your duties in the endoscopy unit			
Diagnostic	17 (14.3%)	1 (4.8%)	0.004
Advanced therapeutics	31 (26.1%)	13 (61.9%)	
Basic therapeutics	71 (59.7%)	7 (33.3%)	
Endoscopy unit			
Outpatient	14 (11.8%)	1 (4.8%)	0.543
Hospital	9 (7.6%)	1 (4.8%)	
Mixed	96 (80.7%)	19 (90.5%)	
Is your unit a university unit and/or is it an endoscopy training center?			
No	75 (63.0%)	11 (52.4%)	0.496
Yes	44 (37.0%)	10 (47.6%)	
Type of funding of your endoscopy unit			
Mostly private	66 (55.5%)	14 (66.7%)	0.473
Mostly Public or Governmental	53 (44.5%)	7 (33.3%)	
Do you apply surveys or other instruments to measure user experience and/or satisfaction in your endoscopy unit?			
No	70 (58.8%)	7 (33.3%)	0.002
I do not know	20 (16.8%)	1 (4.8%)	
Yes	29 (24.4%)	13 (61.9%)	
Do you have a support team or professional responsible for the continuous improvement in your endoscopy unit or institution?			
No	57 (47.9%)	7 (33.3%)	0.255
I do not know	22 (18.5%)	3 (14.3%)	
Yes	40 (33.6%)	11 (52.4%)	
Are patients contacted before procedures to solve questions? (e.g. by phone calls, mobile applications, email or other)			
No	37 (31.1%)	3 (14.3%)	0.129
I do not know	6 (5.0%)	0 (0%)	
Yes	76 (63.9%)	18 (85.7%)	
Do you have local or standardized protocols for sedation and/or analgesia for endoscopic procedures?			
No	52 (43.7%)	7 (33.3%)	0.275
I do not know	7 (5.9%)	0 (0%)	
Yes	60 (50.4%)	14 (66.7%)	
Regarding anesthesia assistance during endoscopic procedures			
We have medical anesthesia equipment	90 (75.6%)	20 (95.2%)	0.0835
We do not have medical anesthesia equipment	29 (24.4%)	1 (4.8%)	
Does your endoscopy unit get in contact with all patients who underwent polypectomy within 1 week after the endoscopic procedure to resolve questions and/or to detect complications?			
No	86 (72.3%)	15 (71.4%)	0.707
I do not know	17 (14.3%)	2 (9.5%)	
Yes	16 (13.4%)	4 (19.0%)	
Does your endoscopy unit get in contact or follow up with patients who have an adverse event during an endoscopic procedure?			
No	33 (27.7%)	5 (23.8%)	0.118
I do not know	26 (21.8%)	1 (4.8%)	
Yes	60 (50.4%)	15 (71.4%)	
Who performs the informed consent procedure before an endoscopic procedure?			
Medical doctor	86 (72.3%)	15 (71.4%)	1.00
Other health professional	33 (27.7%)	6 (28.6%)	
Does your endoscopic unit report most adverse events by a recording system?			
No	14 (11.8%)	2 (9.5%)	0.156
I do not know	17 (14.3%)	0 (0%)	
Yes	88 (73.9%)	19 (90.5%)	
Regarding existing medical liability insurance and/or legal advice, which one do you have?			
None	6 (5.0%)	1 (4.8%)	1.00
FALMED insurance or other	113 (95.0%)	20 (95.2%)	

### Medical professional liability insurance

Table 3 shows the association between general characteristics of participants and self-reports of malpractice insurance. Of participants, 95% reported at least one MPL insurance, with no differences by gender. Insurance status was not associated with report of complaints or malpractice lawsuits. Only the role in the endoscopy unit was associated with MPL insurance status ( *P* = 0.048).

**Table:** **Table 3** Association between general characteristics of participants and self-reporting of medical liability insurance.

	None (n = 7)	FALMED insurance or other (n = 133)	*P* value
Age			
Mean (SD)	52.7 (14.3)	48.6 (11.1)	0.480
Median [min, max]	49.0 [35.0, 74.0]	45.0 [31.0, 76.0]	
Missing	0 (0%)	4 (3.0%)	
Gender			
Female	2 (28.6%)	39 (29.3%)	1.00
Male	5 (71.4%)	94 (70.7%)	
Medical specialty			
Surgery	3 (42.9%)	30 (22.6%)	0.277
Gastroenterology	3 (42.9%)	93 (69.9%)	
Other	1 (14.3%)	5 (3.8%)	
Pediatrics	0 (0%)	5 (3.8%)	
Nationality			
Chilean	7 (100%)	123 (92.5%)	0.989
Ecuadorian	0 (0%)	1 (0.8%)	
Venezuelan	0 (0%)	1 (0.8%)	
Bolivian	0 (0%)	1 (0.8%)	
Colombian	0 (0%)	1 (0.8%)	
Hungarian	0 (0%)	6 (4.5%)	
Years of practice			
Between 5 to 10 years	2 (28.6%)	39 (29.3%)	0.427
More than 10 years	3 (42.9%)	78 (58.6%)	
Less than 5 years	2 (28.6%)	16 (12.0%)	
Have you completed a formal training program in digestive endoscopy?			
No	1 (14.3%)	7 (5.3%)	0.867
Yes	6 (85.7%)	126 (94.7%)	
Your role in the endoscopy unit: Select all the alternatives that correspond to your duties in the endoscopy unit			
Diagnostic	3 (42.9%)	15 (11.3%)	0.048
Advanced therapeutics	1 (14.3%)	43 (32.3%)	
Basic therapeutics	3 (42.9%)	75 (56.4%)	
Endoscopy unit			
Outpatient	2 (28.6%)	13 (9.8%)	0.244
Hospital	5 (71.4%)	110 (82.7%)	
Mixed	0 (0%)	10 (7.5%)	
Is your unit a university unit and/or is it an endoscopy training center?			
No	6 (85.7%)	80 (60.2%)	0.339
Yes	1 (14.3%)	53 (39.8%)	
Type of funding of your endoscopy unit			
Mostly private	3 (42.9%)	77 (57.9%)	0.695
Mostly Public or Governmental	4 (57.1%)	56 (42.1%)	
Do you apply surveys or other instruments to measure user experience and/or satisfaction in your endoscopy unit?			
No	6 (85.7%)	71 (53.4%)	0.226
I do not know	1 (14.3%)	41 (30.8%)	
Yes	0 (0%)	21 (15.8%)	
Do you have a support team or professional responsible for the continuous improvement in your endoscopy unit or institution?			
No	2 (28.6%)	62 (46.6%)	0.498
I do not know	1 (14.3%)	24 (18.0%)	
Yes	4 (57.1%)	47 (35.3%)	
Are patients contacted before procedures to solve questions? (e.g. by phone calls, mobile applications, email or other)			
No	1 (14.3%)	39 (29.3%)	0.543
I do not know	6 (85.7%)	88 (66.2%)	
Yes	0 (0%)	6 (4.5%)	
Do you have local or standardized protocols for sedation and/or analgesia for endoscopic procedures?			
No	4 (57.1%)	55 (41.4%)	0.637
I do not know	3 (42.9%)	71 (53.4%)	
Yes	0 (0%)	7 (5.3%)	
Regarding anesthesia assistance during endoscopic procedures			
We have medical anesthesia equipment	6 (85.7%)	104 (78.2%)	1.00
We do not have medical anesthesia equipment	1 (14.3%)	29 (21.8%)	
Does your endoscopy unit get in contact with all patients who underwent polypectomy within 1 week after the endoscopic procedure to resolve questions and/or to detect complications?			
No	6 (85.7%)	95 (71.4%)	0.537
I do not know	1 (14.3%)	18 (13.5%)	
Yes	0 (0%)	20 (15.0%)	
Does your endoscopy unit get in contact or follow up with patients who have an adverse event during an endoscopic procedure?			
No	4 (57.1%)	34 (25.6%)	0.134
I do not know	3 (42.9%)	72 (54.1%)	
Yes	0 (0%)	27 (20.3%)	
Who performs the informed consent procedure before an endoscopic procedure?			
Medical doctor	3 (42.9%)	98 (73.7%)	0.180
Other health professional	4 (57.1%)	35 (26.3%)	
Does your endoscopic unit report most adverse events by a recording system?			
No	2 (28.6%)	14 (10.5%)	0.320
I do not know	1 (14.3%)	16 (12.0%)	
Yes	4 (57.1%)	103 (77.4%)	
Have you received complaints from patients and/or family members during your endoscopic practice?			
No	3 (42.9%)	60 (45.1%)	1.00
Yes	4 (57.1%)	73 (54.9%)	

## Discussion

To the best of our knowledge, this is the first study exploring the medicolegal experience of endoscopists in Latin America. Complaints and malpractice lawsuits are an increasing reality across modern medical practice 1 19 20 . This scenario impacts patient care including deterioration of the physician-patient relationship and influencing clinical decisions, which in turn leads to increased healthcare usage 2 .

The results of our study revealed that 55.1% of participants surveyed reported at least one complaint, with an average of 1.5 complaints per year. In addition, 14.5% of survey respondents reported at least one endoscopic procedure that resulted in a lawsuit alleging malpractice.

Our study highlights that colonoscopy was the most frequent procedure associated with complaints and lawsuits. This finding is not surprising because colonoscopy is a more frequent procedure than other procedures (e.g. ERCP), and it is consistent with findings reported by U.S. publications 6 7 21 . However, professionals performing advanced therapeutic endoscopy report more complaints and lawsuits compared with those who do not perform such procedures. This may be explained by a higher risk of AEs associated with advanced therapeutic endoscopy, particularly ERCP 22 .

Also, our study emphasizes that AEs and sedation are key clinical aspects that frequently lead to complaints. On one hand, AEs are preventable but cannot be eliminated, they are more frequent and severe during therapeutic procedures, and they do not necessarily imply negligence or malpractice. Risk of AEs has changed with development of new techniques and devices, which has expanded the list of indications and safety of endoscopic procedures. Perforation, bleeding, pancreatitis, and death were the most frequently reported causes of complaints in our study. Therefore, it is crucial that endoscopist training curricula include diagnosis and management of AEs, as recommended by international guidelines 23 24 25 26 27 .

Other strategies for improvement are ongoing measurement of multiple quality indicators and fostering a culture of constructive feedback among physicians aimed at learning within an endoscopy unit 28 29 . Standardized record systems are necessary because detecting areas of improvement and changes in protocols afterwards are probably the best prevention strategies. In our study, assessment of the user experience and/or satisfaction was associated with a higher risk of complaints and lawsuits. This may be explained by reverse causality, that is, endoscopy units performing more complex procedures and with a higher risk of AEs are the ones that have established better monitoring and recording systems.

In contrast, sedation presents a complex challenge: Although minimal sedation in procedures such as EGD and colonoscopy may be safer from a cardiorespiratory perspective, it is not necessarily so from a medicolegal standpoint due to increasing patient expectations regarding pain management and the overall procedure experience. This underscores the need to develop personalized local sedation protocols that balance safety and patient satisfaction 30 .

It was expected that physicians with more years of endoscopic practice would face more complaints and lawsuits, because they have been performing endoscopic procedures longer, as described by Adams et al. (2019) 2 . However, this extended experience may also act as a protective factor, enabling timely detection and appropriate treatment of AEs. Similarly, the higher percentage of self-reported complaints and lawsuits among male and Chilean physicians could be attributed to their greater representation within the sample. Nonetheless, sex-based differences observed in surgical practice underscore the need for further studies specifically designed to explore this issue in endoscopy 31 .

Approximately 30% of respondents indicated that communication between physicians and patients was one of the causes of complaints. Providing precise, empathetic, and timely information to patients and/or families can prevent subsequent conflicts and should be understood as a key component of professional practice of endoscopists. In recent years, patient experience and satisfaction during medical procedures have gained interest 12 .

The informed consent process is essential because it is the cornerstone of ethical medical practice, procedures, and treatments 32 33 . Informed consent is a medical responsibility; however, our study revealed that in almost 25% of the endoscopy units, the informed consent process was performed by other professionals. It would be ideal to use specific informed consent documents for endoscopic procedures containing precise and updated information detailing the benefits and risks of the procedures.

Although prosecution of medical malpractice contributes to protect patient rights and improve quality of medical care, it may lead physicians to recommend unnecessary examinations and procedures to protect themselves from potential lawsuits, a practice known as “defensive medicine” 4 34 35 . It may also lead physicians to avoid essential but high-risk procedures from a MPL point of view.

Our results also indicate that costs and administrative situations are also leading causes of complaints. Clear communication of costs to patients before endoscopic procedures and adherence to schedules are opportunities for improvement for endoscopy unit organization. Furthermore, adequate personnel are essential to ensure patient safety, quality of the endoscopic procedure, and user satisfaction.

**Fig. 3:**
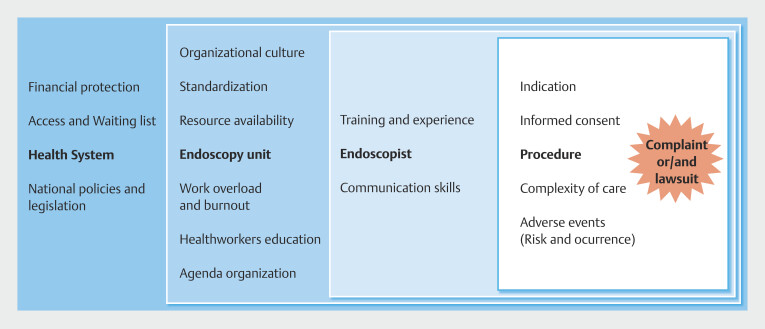
Conceptual model of determinants of MPL complaints and lawsuit in digestive endoscopy.

Based on the results of our study, we propose a conceptual model of the determinants of complaints and lawsuits. This model permits identification of areas for action ( Fig. 3 ). Although the origin of medicolegal situations can be traced to a distal level of the healthcare system and existing local legislation, our study focuses on the nearest and potentially modifiable factors by endoscopists.

This study presents several limitations. The design and the sensitive nature of the research topic may have introduced memory and information biases, respectively. In addition, this survey did not distinguish whether lawsuits were civil or criminal. Furthermore, we did not explore the emotional impact of complaints and lawsuits on endoscopists, which could potentially discourage them from performing high-risk procedures, such as therapeutic endoscopy. Finally, only Chilean-based endoscopists participated. However, similar results are expected from other Latin American countries because our healthcare systems share similar organizational and financial challenges.

## Conclusions

In conclusion, gastrointestinal endoscopists are frequently exposed to complaints and lawsuits. Key factors include procedure costs, AEs, sedation practices, years of experience, type of endoscopic procedure, and communication. To minimize medicolegal risks, healthcare professionals must critically evaluate and enhance their practices, emphasizing adherence to a well-defined lex artis. This should encompass appropriate medical indications, clear and empathetic communication, thorough informed consent processes, and meticulous documentation.

Such measures not only reduce the likelihood of litigation but also safeguard against defensive medical practices motivated by fear of legal consequences. Ultimately, the most effective legal protection for physicians lies in upholding the principles of evidence-based, patient-centered care within a robust lex artis framework.

## Supplementary Material

Supplementary Material Zusatzmaterial

## References

[1] Kantrowitz MG Medical malpractice and gastrointestinal endoscopy Curr Opin Gastroenterol 2022 38 467 471 10.1097/MOG.0000000000000863 35881965

[2] Adams MA Allen JI Medical professional liability in gastroenterology: Understanding the claims landscape and proposed mechanisms for reform Clin Gastroenterol Hepatol 2019 17 2392 23960 10.1016/j.cgh.2019.07.002 31279950

[3] Jena AB Seabury S Lakdawalla D Malpractice risk according to physician specialty N Engl J Med 2011 365 629 636 10.1056/NEJMsa1012370 21848463 PMC3204310

[4] Azizian J Dalai C Adams MA Medical professional liability in gastroenterology: definitions, trends, risk factors, provider behaviors, and implications Expert Rev Gastroenterol Hepatol 2021 15 909 918 10.1080/17474124.2021.1940957 34112036

[5] Sheikh K Webster G Endoscopy-related litigation in UK: causes, costs and outcome Gut 2010 59 A56

[6] Panuganti PL Hartnett DA Eltorai AEM Colorectal Cancer Litigation: 1988–2018 Am J Gastroenterol 2020 115 1525 1531 10.14309/ajg.0000000000000705 32453040

[7] Patel KS Kothari P Gantz O Current trends and predictors of case outcomes for malpractice in colonoscopy in the United States J Clin Gastroenterol 2022 56 49 54 10.1097/MCG.0000000000001471 33337638

[8] Rubenstein JH Saini SD Kuhn L Influence of malpractice history on the practice of screening and surveillance for Barrett’s esophagus Am J Gastroenterol 2008 103 842 849 10.1111/j.1572-0241.2007.01689.x 18076733

[9] Hiyama T Tanaka S Yoshihara M Medical malpractice litigation related to gastrointestinal endoscopy in Japan: a two-decade review of civil court cases World J Gastroenterol 2006 12 6857 6860 10.3748/wjg.v12.i42.6857 17106936 PMC4087442

[10] Everett SM Triantafyllou K Hassan C Informed consent for endoscopic procedures: European Society of Gastrointestinal Endoscopy (ESGE) Position Statement Endoscopy 2023 55 952 966 10.1055/a-2133-3365 37557899

[11] ASGE Endoscopy Unit Quality Indicator Taskforce Day LW Cohen J Quality indicators for gastrointestinal endoscopy units VideoGIE 2017 2 119 140 29905282 10.1016/j.vgie.2017.02.007 PMC5990988

[12] Day LW Savides TJ Patient Experience in the Gastrointestinal Endoscopy Unit Clin Gastroenterol Hepatol 2022 20 723 726 10.1016/j.cgh.2021.12.001 34864159

[13] Brown S Bevan R Rubin G Patient-derived measures of GI endoscopy: a meta-narrative review of the literature Gastrointest Endosc 2015 81 1130 1140.e1-e9 25864891 10.1016/j.gie.2014.11.047

[14] Valori R Cortas G de Lange T Performance measures for endoscopy services: A European Society of Gastrointestinal Endoscopy (ESGE) quality improvement initiative United European Gastroenterol J 2019 7 21 44 10.1177/2050640618810242 PMC6374839 30788114

[15] López-Picazo J Alberca de Las Parras F Sánchez Del Río A Quality indicators in digestive endoscopy: introduction to structure, process, and outcome common indicators Rev Esp Enferm Dig 2017 109 435 450 28553719 10.17235/reed.2017.5035/2017

[16] Kerdsirichairat T Shin EJ Important quality metrics and standardization in endoscopy Gastrointest Endosc Clin N Am 2021 31 727 742 10.1016/j.giec.2021.05.009 34538412

[17] Rizk MK Sawhney MS Cohen J Quality indicators common to all GI endoscopic procedures Am J Gastroenterol 2015 110 48 59 10.1016/j.gie.2014.07.055 25448874

[18] Ley 20584: Regula los derechos y deberes que tienen las personas en relación con acciones vinculadas a su atención en salud https://www.bcn.cl/leychile/navegar?idNorma=1039348

[19] Verdugo AM, Cáceres C. Impacto económico de la judicialización en Chile. Repositorio Universidad de Chile 2017 https://repositorio.uchile.cl/bitstream/handle/2250/145445/Verdugo%20Marchese%20Anamar%E2%94%9C%C2%A1a.pdf?sequence=1

[20] Suárez FM Demandas por responsabilidad médica en Chile. Análisis de montos, condenas y duración Rev Derecho Esc Postgrado 2015 0 1 28

[21] Hernandez LV Klyve D Regenbogen SE Malpractice claims for endoscopy World J Gastrointest Endosc 2013 5 169 173 10.4253/wjge.v5.i4.169 23596540 PMC3627840

[22] Dumonceau JM Kapral C Aabakken L ERCP-related adverse events: European Society of Gastrointestinal Endoscopy (ESGE) Guideline Endoscopy 2020 52 127 149 10.1055/a-1075-4080 31863440

[23] Waschke KA Anderson J Valori RM ASGE principles of endoscopic training Gastrointest Endosc 2019 90 27 34 10.1016/j.gie.2018.10.017 31122745

[24] Lee JH Kedia P Stavropoulos SN AGA Clinical Practice Update on Endoscopic Management of Perforations in Gastrointestinal Tract: Expert Review Clin Gastroenterol Hepatol 2021 19 2252 226100 34224876 10.1016/j.cgh.2021.06.045

[25] Pimentel-Nunes P Pioche M Albéniz E Curriculum for endoscopic submucosal dissection training in Europe: European Society of Gastrointestinal Endoscopy (ESGE) Position Statement Endoscopy 2019 51 980 992 10.1055/a-0996-0912 31470448

[26] Tate DJ Argenziano ME Anderson J Curriculum for training in endoscopic mucosal resection in the colon: European Society of Gastrointestinal Endoscopy (ESGE) Position Statement Endoscopy 2023 55 645 679 10.1055/a-2077-0497 37285908

[27] Johnson G Webster G Boškoski I Curriculum for ERCP and endoscopic ultrasound training in Europe: European Society of Gastrointestinal Endoscopy (ESGE) Position Statement Endoscopy 2021 53 1071 1087 10.1055/a-1537-8999 34311472

[28] Waddingham W Kamran U Kumar B Complications of diagnostic upper Gastrointestinal endoscopy: common and rare – recognition, assessment and management BMJ Open Gastroenterol 2022 9 e000688 10.1136/bmjgast-2021-000688 PMC9806027 36572454

[29] Sint Nicolaas J de Jonge V de Man RA The Global Rating Scale in clinical practice: a comprehensive quality assurance programme for endoscopy departments Dig Liver Dis 2012 44 919 924 22840567 10.1016/j.dld.2012.06.021

[30] Sidhu R Turnbull D Haboubi H British Society of Gastroenterology guidelines on sedation in gastrointestinal endoscopy Gut 2024 73 219 245 10.1136/gutjnl-2023-330396 37816587 PMC10850688

[31] Wallis CJD Jerath A Aminoltejari K Surgeon sex and long-term postoperative outcomes among patients undergoing common surgeries JAMA Surg 2023 158 1185 1194 37647075 10.1001/jamasurg.2023.3744 PMC10469289

[32] ASGE Standards of Practice Committee Storm AC Fishman DS American Society for Gastrointestinal Endoscopy guideline on informed consent for GI endoscopic procedures Gastrointest Endosc 2022 95 207 21500 34998575 10.1016/j.gie.2021.10.022

[33] Everett SM Triantafyllou K Hassan C Informed consent for endoscopic procedures: European Society of Gastrointestinal Endoscopy (ESGE) Position Statement Endoscopy 2023 55 952 966 10.1055/a-2133-3365 37557899

[34] Miziara ID Miziara CSMG Medical errors, medical negligence and defensive medicine: A narrative review Clinics (Sao Paulo) 2022 77 100053 10.1016/j.clinsp.2022.100053 PMC9160317 35640458

[35] Ries NM Jansen J Physicians’ views and experiences of defensive medicine: An international review of empirical research Health Policy 2021 125 634 642 10.1016/j.healthpol.2021.02.005 33676778

