# Supplementary material for: Medicolegal aspects of digestive endoscopy: Results of a Chilean national survey
Source: Endosc Int Open. 2025 May 12;13:a25706490. doi: 10.1055/a-2570-6490 (PMC12080522; doi:10.1055/a-2570-6490)
Supplement: Supplementary file 1 — Supplementary Material [file 10-1055-a-2570-6490_25717182.pdf]

Supplementary material

NATIONAL SURVEY OF MEDICOLEGAL ASPECTS OF DIGESTIVE ENDOSCOPY

1.General information

Age: ______

Gender: ______

Medical specialty
- Gastroenterology
- Surgery
- Other
- Pediatrics

Nationality
- Chilean
- Other

Years of practice
- Between 5 to 10 years
- More than 10 years
- Less than 5 years

Have you completed a formal training program in digestive endoscopy?
- Yes
- No

Your role in the endoscopy unit: Select all the alternatives that correspond to your duties in the endoscopy unit
- Coordination or directive position
- Diagnostic
- Advanced therapeutics
- Basic therapeutics

2. Endoscopy Unit characteristic

Endoscopy unit setting
- Outpatient
- Hospital

- Mixed

Is your unit a university unit and/or is it an endoscopy training center?
- Yes
- No

Type of funding of your endoscopy unit
- Mostly private
- Mostly public or governmental

Do you apply surveys or other instruments to measure user experience and/or satisfaction in your endoscopy unit?
- No
- I do not know
- Yes

Do you have a support team or professional responsible for the continuous improvement in your endoscopy unit or institution?
- No
- I do not know
- Yes

Are patients contacted before procedures to solve questions? (e.g. by phone calls, mobile applications, email or other)
- No
- I do not know
- Yes

Do you have local or standardized protocols for sedation and/or analgesia for endoscopic procedures?
- No
- I do not know
- Yes

Regarding anesthesia assistance during endoscopic procedures
- We have medical anesthesia equipment
- We do not have medical anesthesia equipment

Does your endoscopy unit get in contact with all patients who underwent polypectomy within one week after the endoscopic procedure to resolve questions and/or to detect complications?

- No
- I do not know
- Yes

Does your endoscopy unit get in contact or follow up with patients who have an adverse event during an endoscopic procedure?

- No
- I do not know
- Yes

Who performs the informed consent procedure before an endoscopic procedure?

- Medical doctor
- Other health professional

Does your endoscopic unit report most adverse events by a recording system?

- No
- I do not know
- Yes

<u>3. Medicolegal experience</u>

Who is responsible for obtaining informed consent from the patient in your unit?

- Endoscopist or fellow
- Nurse team
- Another team member

Which kind of informed consent form does your unit use?

- Universal or standard informed consent form (for any kind of procedures, not only digestive endoscopy)
- A specific informed consent form (only for digestive endoscopy)

Has the claims rate in your endoscopy unit changed over the last 3 years?

- Increase
- No change
- Decrease
- I do not know

Have you ever received claims from patients and/or family members?
- No
- Yes

If you have received claims from patients and/or family members, how many claims do you receive per year (mean)?

____

In your experience, which has been the leading procedure that motivates claims? (You can choose one or more)
- Diagnostic upper gastrointestinal endoscopy (esophagogastroduodenoscopy)
- Diagnostic colonoscopy
- Polypectomy
- Endoscopic mucosal resection or endoscopic submucosal dissection
- Endoscopic hemostasis
- Endoscopic foreign body removal
- Percutaneous endoscopic gastrostomy
- Endoscopic endoprosthesis insertion
- Endoscopic nasogastric tube insertion
- Endoscopic retrograde cholangiopancreatography
- Endoscopic ultrasound
- Peroral endoscopic myotomy
- Small bowel capsule endoscopy and enteroscopy
- Other

Have you ever received any lawsuits for claimed malpractice from patients and/or family members?
- No
- Yes

In your experience, which has been the leading motivation for a claim or lawsuit? (You can choose one or more)
- Informed consent: Lack, poorly completed or not performed
- Poor communication with the patient and/or family members
- Delay or suspension in the agenda
- Problems related to the costs of the procedure
- Adverse event: failure in recognition and/or management
- Deficit in infrastructure, equipment, or supplies.
- Deficit in human resources: work overload, burnout
- Lack of experience, competence or training in the procedure performed

- Inadequate indication
- Patient`s experience with sedation
- Patient`s experience with the complete process (example, facilities, accommodation, manners of health workers)
- Diagnostic error
- Other

What are the adverse events that motivate claims from patients and/or family members? (You can choose one or more)

- Bleeding
- Perforation
- Infection
- Pancreatitis
- Embolism
- Death
- Other

Regarding existing medical liability insurance and/or legal advice, which one do you have?

- None
- FALMED insurance or other
